# 3-(4-*tert*-Butyl­phen­yl)-1-(4-fluoro­phen­yl)-3-hydroxy­prop-2-en-1-one

**DOI:** 10.1107/S1600536808042372

**Published:** 2008-12-17

**Authors:** Chunyang Zheng, Dunjia Wang, Ling Fan

**Affiliations:** a Hubei Key Laboratory of Bioanalytical Techniques, Hubei Normal University, Huangshi 435002, People’s Republic of China; b College of Chemistry and Environmental Engineering, Hubei Normal University, Huangshi 435002, People’s Republic of China

## Abstract

The title mol­ecule, C_19_H_19_FO_2_, exits in the enol form with a dihedral angle of 33.06 (8)° between the two benzene rings. The mol­ecular conformation is stabilized in part by an intra­molecular O—H⋯O hydrogen bond.

## Related literature

For background information on 1,3-diketones, see: Baskar & Roesky (2005 ▶); Bassett *et al.* (2004 ▶); Bertolasi *et al.* (1991 ▶); Jang *et al.* (2006 ▶); Soldatov *et al.* (2003 ▶); Vila *et al.* (1991 ▶).
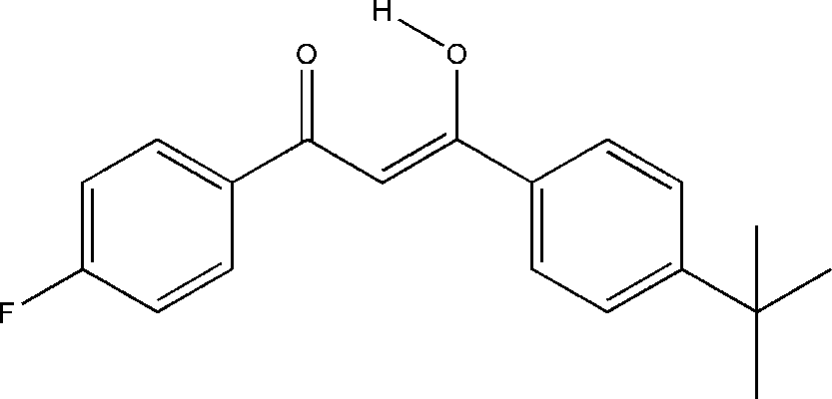


## Experimental

### 

#### Crystal data

C_19_H_19_FO_2_
*M* *_r_* = 298.34
Monoclinic, 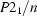

*a* = 9.8349 (12) Å
*b* = 10.0163 (13) Å
*c* = 16.232 (2) Å
β = 97.788 (2)°
*V* = 1584.3 (3) Å^3^
*Z* = 4
Mo *K*α radiation
μ = 0.09 mm^−1^
*T* = 298 (2) K
0.20 × 0.10 × 0.10 mm

#### Data collection

Bruker SMART CCD diffractometer
Absorption correction: multi-scan (*SADABS*; Sheldrick, 1996 ▶) *T*_min_ = 0.993, *T*_max_ = 0.995
12039 measured reflections
3099 independent reflections
2199 reflections with *I* > 2σ(*I*)
*R*_int_ = 0.074

#### Refinement

*R*[*F* ^2^ > 2σ(*F* ^2^)] = 0.050
*wR*(*F* ^2^) = 0.131
*S* = 1.00
3099 reflections
205 parameters
H atoms treated by a mixture of independent and constrained refinement
Δρ_max_ = 0.17 e Å^−3^
Δρ_min_ = −0.19 e Å^−3^

### 

Data collection: *SMART* (Bruker, 1997 ▶); cell refinement: *SAINT* (Bruker, 1999 ▶); data reduction: *SAINT*; program(s) used to solve structure: *SHELXS97* (Sheldrick, 2008 ▶); program(s) used to refine structure: *SHELXL97* (Sheldrick, 2008 ▶); molecular graphics: *SHELXTL* (Sheldrick, 2008 ▶); software used to prepare material for publication: *PLATON* (Spek, 2003 ▶).

## Supplementary Material

Crystal structure: contains datablocks global, I. DOI: 10.1107/S1600536808042372/lh2743sup1.cif

Structure factors: contains datablocks I. DOI: 10.1107/S1600536808042372/lh2743Isup2.hkl

Additional supplementary materials:  crystallographic information; 3D view; checkCIF report

## Figures and Tables

**Table 1:** Hydrogen-bond geometry (Å, °)

*D*—H⋯*A*	*D*—H	H⋯*A*	*D*⋯*A*	*D*—H⋯*A*
O2—H2*A*⋯O1	1.16 (2)	1.38 (2)	2.4720 (16)	154 (2)

**Fig. 1.**
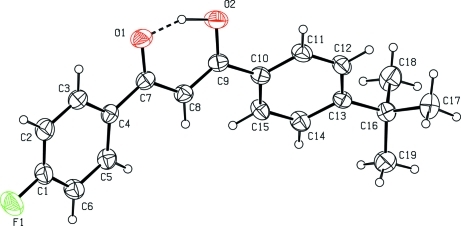
The molecular structure of (I). Displacement ellipsoids are drawn at the 50% probability level. The dashed line indicates a intramolecular hydrogen bond.

## supplementary crystallographic information

### Comment

1,3-Diketones are interesting due to their enolic tautomeric forms and their ability to form strong intermolecular or intramolecular hydrogen bonds (Bertolasi *et al.*, 1991; Vila *et al.*, 1991). They are used widely in the chemistry of metallocomplexes (Baskar *et al.*, 2005; Bassett *et al.*, 2004; Jang *et al.*, 2006; Soldatov *et al.*, 2003). The title compound (I) (Fig. 1), is in the enol form stabilized by an intramolecular O-H···O hydrogen bond (see Table 1).

### Experimental

1-(4-fluorophenyl)ethanone (1.38 g, 0.01 mol), methyl 4-*tert*-butylbenzoate (1.92 g, 0.01 mol), NaNH_2_ (0.78 g, 0.02 mol) and dry ether (60 ml) were placed into round bottom flask. The mixture was stirred for 6 h at room temperature under a blanket of nitrogen, acidified with dilute hydrochloric acid, and stirring was continued until all solids dissolved. The ether layer was separated and washed with saturated NaHCO_3_ solution, dried over anhydrous Na_2_SO_4_ and was removed by evaporation. The residual solid was recrystallized from ethanol solution to give the title compound (I) (yield 1.78 g, 59.6%, m.p. 388 K). Crystals suitable for X-ray diffraction were grown by slow evaporation of a CHCl_3_—EtOH (1:4) solution of the title compound at room temperature.

### Refinement

H atoms were placed in geometrically idealized positions and constrained to ride on their parent atoms, with C—H distances of 0.93 to 0.96 Å, and with *U*_iso_(H) = 1.2 *U*_eq_(C). The H atom of the hydroxyl group was located in a difference Fourier map and its position was refined freely, with *U*_iso_(H) = 1.5 *U*_iso_(O).

### Crystal data

**Table:**

C_19_H_19_FO_2_	*F*(000) = 632
*M**_r_* = 298.34	*D*_x_ = 1.251 Mg m^−^^3^
Monoclinic, *P*2_1_/*n*	Melting point: 388 K
Hall symbol: -P 2yn	Mo *K*α radiation, λ = 0.71073 Å
*a* = 9.8349 (12) Å	Cell parameters from 3223 reflections
*b* = 10.0163 (13) Å	θ = 2.3–22.9°
*c* = 16.232 (2) Å	µ = 0.09 mm^−^^1^
β = 97.788 (2)°	*T* = 298 K
*V* = 1584.3 (3) Å^3^	Block, colorless
*Z* = 4	0.20 × 0.10 × 0.10 mm

### Data collection

**Table:**

Bruker SMART CCD diffractometer	3099 independent reflections
Radiation source: fine-focus sealed tube	2199 reflections with *I* > 2σ(*I*)
graphite	*R*_int_ = 0.074
φ and ω scans	θ_max_ = 26.0°, θ_min_ = 2.3°
Absorption correction: multi-scan (*SADABS*; Sheldrick, 1996)	*h* = −11→12
*T*_min_ = 0.993, *T*_max_ = 0.995	*k* = −12→12
12039 measured reflections	*l* = −19→19

### Refinement

**Table:**

Refinement on *F*^2^	Primary atom site location: structure-invariant direct methods
Least-squares matrix: full	Secondary atom site location: difference Fourier map
*R*[*F*^2^ > 2σ(*F*^2^)] = 0.050	Hydrogen site location: inferred from neighbouring sites
*wR*(*F*^2^) = 0.131	H atoms treated by a mixture of independent and constrained refinement
*S* = 1.00	*w* = 1/[σ^2^(*F*_o_^2^) + (0.07*P*)^2^] where *P* = (*F*_o_^2^ + 2*F*_c_^2^)/3
3099 reflections	(Δ/σ)_max_ = 0.001
205 parameters	Δρ_max_ = 0.17 e Å^−^^3^
0 restraints	Δρ_min_ = −0.18 e Å^−^^3^

### Special details

**Table:**

Geometry. All e.s.d.'s (except the e.s.d. in the dihedral angle between two l.s. planes) are estimated using the full covariance matrix. The cell e.s.d.'s are taken into account individually in the estimation of e.s.d.'s in distances, angles and torsion angles; correlations between e.s.d.'s in cell parameters are only used when they are defined by crystal symmetry. An approximate (isotropic) treatment of cell e.s.d.'s is used for estimating e.s.d.'s involving l.s. planes.
Refinement. Refinement of *F*^2^ against ALL reflections. The weighted *R*-factor *wR* and goodness of fit *S* are based on *F*^2^, conventional *R*-factors *R* are based on *F*, with *F* set to zero for negative *F*^2^. The threshold expression of *F*^2^ > σ(*F*^2^) is used only for calculating *R*-factors(gt) *etc*. and is not relevant to the choice of reflections for refinement. *R*-factors based on *F*^2^ are statistically about twice as large as those based on *F*, and *R*- factors based on ALL data will be even larger.

### Fractional atomic coordinates and isotropic or equivalent isotropic displacement parameters (Å^2^)

**Table:**

	*x*	*y*	*z*	*U*_iso_*/*U*_eq_	
C1	0.12996 (18)	0.09274 (19)	−0.07276 (10)	0.0599 (5)	
C2	0.0803 (2)	0.2198 (2)	−0.08119 (10)	0.0666 (5)	
H2	0.0522	0.2559	−0.1335	0.080*	
C3	0.07296 (18)	0.29335 (17)	−0.01033 (9)	0.0587 (5)	
H3	0.0401	0.3805	−0.0150	0.070*	
C4	0.11376 (15)	0.23969 (16)	0.06796 (9)	0.0457 (4)	
C5	0.16498 (16)	0.11083 (17)	0.07303 (9)	0.0545 (4)	
H5	0.1943	0.0741	0.1250	0.065*	
C6	0.17340 (17)	0.03602 (18)	0.00290 (10)	0.0596 (5)	
H6	0.2076	−0.0506	0.0068	0.071*	
C7	0.09992 (16)	0.32242 (16)	0.14225 (9)	0.0491 (4)	
C8	0.11727 (16)	0.27023 (16)	0.22333 (9)	0.0499 (4)	
H8	0.1464	0.1824	0.2321	0.060*	
C9	0.09179 (16)	0.34703 (16)	0.29012 (9)	0.0498 (4)	
C10	0.09963 (16)	0.29479 (16)	0.37555 (9)	0.0475 (4)	
C11	0.01481 (18)	0.34617 (16)	0.42948 (10)	0.0561 (4)	
H11	−0.0459	0.4148	0.4118	0.067*	
C12	0.01961 (17)	0.29659 (17)	0.50883 (10)	0.0565 (4)	
H12	−0.0396	0.3319	0.5432	0.068*	
C13	0.10956 (15)	0.19580 (15)	0.53959 (9)	0.0464 (4)	
C14	0.19521 (17)	0.14742 (17)	0.48502 (9)	0.0557 (4)	
H14	0.2580	0.0808	0.5032	0.067*	
C15	0.19034 (17)	0.19472 (17)	0.40495 (9)	0.0541 (4)	
H15	0.2488	0.1589	0.3702	0.065*	
C16	0.11634 (16)	0.14446 (16)	0.62880 (9)	0.0512 (4)	
C17	−0.02548 (19)	0.1488 (2)	0.65807 (11)	0.0744 (6)	
H17A	−0.0544	0.2400	0.6614	0.112*	
H17B	−0.0205	0.1080	0.7119	0.112*	
H17C	−0.0903	0.1012	0.6193	0.112*	
C18	0.2134 (2)	0.2360 (2)	0.68505 (10)	0.0774 (6)	
H18A	0.3020	0.2364	0.6664	0.116*	
H18B	0.2221	0.2039	0.7412	0.116*	
H18C	0.1770	0.3250	0.6827	0.116*	
C19	0.1691 (2)	0.00150 (18)	0.63681 (12)	0.0791 (6)	
H19A	0.1140	−0.0541	0.5973	0.119*	
H19B	0.1639	−0.0305	0.6920	0.119*	
H19C	0.2628	−0.0010	0.6262	0.119*	
F1	0.13561 (14)	0.01881 (12)	−0.14199 (6)	0.0933 (4)	
O1	0.06597 (13)	0.44469 (12)	0.12968 (7)	0.0671 (4)	
O2	0.05431 (14)	0.47163 (12)	0.27994 (8)	0.0720 (4)	
H2A	0.050 (2)	0.485 (2)	0.2088 (15)	0.108*	

### Atomic displacement parameters (Å^2^)

**Table:**

	*U*^11^	*U*^22^	*U*^33^	*U*^12^	*U*^13^	*U*^23^
C1	0.0655 (12)	0.0679 (12)	0.0478 (9)	0.0042 (9)	0.0136 (8)	−0.0004 (8)
C2	0.0872 (14)	0.0695 (13)	0.0436 (9)	0.0131 (10)	0.0110 (8)	0.0131 (8)
C3	0.0709 (12)	0.0531 (10)	0.0526 (10)	0.0069 (9)	0.0105 (8)	0.0105 (8)
C4	0.0383 (9)	0.0509 (10)	0.0480 (9)	−0.0008 (7)	0.0060 (6)	0.0057 (7)
C5	0.0546 (10)	0.0619 (11)	0.0458 (9)	0.0075 (8)	0.0026 (7)	0.0083 (8)
C6	0.0628 (12)	0.0584 (11)	0.0574 (10)	0.0130 (9)	0.0080 (8)	0.0037 (8)
C7	0.0445 (9)	0.0480 (10)	0.0540 (9)	−0.0026 (7)	0.0035 (7)	0.0054 (7)
C8	0.0547 (10)	0.0466 (10)	0.0475 (9)	0.0040 (8)	0.0040 (7)	0.0024 (7)
C9	0.0493 (10)	0.0455 (10)	0.0525 (9)	−0.0028 (7)	−0.0003 (7)	−0.0013 (7)
C10	0.0484 (9)	0.0456 (9)	0.0468 (8)	−0.0007 (7)	0.0004 (7)	−0.0062 (7)
C11	0.0625 (11)	0.0490 (10)	0.0560 (10)	0.0152 (8)	0.0049 (8)	0.0000 (7)
C12	0.0612 (11)	0.0559 (11)	0.0537 (10)	0.0124 (9)	0.0128 (8)	−0.0052 (8)
C13	0.0465 (9)	0.0443 (9)	0.0475 (8)	−0.0021 (7)	0.0028 (7)	−0.0069 (7)
C14	0.0547 (10)	0.0603 (11)	0.0507 (9)	0.0163 (8)	0.0017 (7)	0.0021 (7)
C15	0.0531 (10)	0.0615 (11)	0.0480 (9)	0.0132 (8)	0.0075 (7)	−0.0049 (7)
C16	0.0502 (10)	0.0554 (10)	0.0471 (9)	−0.0006 (8)	0.0029 (7)	−0.0021 (7)
C17	0.0699 (13)	0.0938 (15)	0.0614 (11)	−0.0013 (11)	0.0153 (9)	0.0105 (10)
C18	0.0857 (14)	0.0914 (15)	0.0515 (10)	−0.0224 (12)	−0.0039 (9)	−0.0023 (9)
C19	0.1093 (17)	0.0651 (13)	0.0644 (12)	0.0161 (12)	0.0167 (11)	0.0115 (9)
F1	0.1407 (12)	0.0884 (9)	0.0527 (6)	0.0260 (7)	0.0201 (6)	−0.0074 (5)
O1	0.0956 (10)	0.0484 (7)	0.0566 (7)	0.0054 (7)	0.0083 (6)	0.0085 (5)
O2	0.1083 (11)	0.0459 (7)	0.0598 (8)	0.0109 (7)	0.0044 (7)	−0.0007 (5)

### Geometric parameters (Å, °)

**Table:**

C1—F1	1.3532 (19)	C11—H11	0.9300
C1—C2	1.364 (3)	C12—C13	1.390 (2)
C1—C6	1.368 (2)	C12—H12	0.9300
C2—C3	1.376 (2)	C13—C14	1.390 (2)
C2—H2	0.9300	C13—C16	1.530 (2)
C3—C4	1.388 (2)	C14—C15	1.378 (2)
C3—H3	0.9300	C14—H14	0.9300
C4—C5	1.384 (2)	C15—H15	0.9300
C4—C7	1.485 (2)	C16—C19	1.523 (2)
C5—C6	1.375 (2)	C16—C18	1.533 (2)
C5—H5	0.9300	C16—C17	1.534 (2)
C6—H6	0.9300	C17—H17A	0.9600
C7—O1	1.2784 (19)	C17—H17B	0.9600
C7—C8	1.405 (2)	C17—H17C	0.9600
C8—C9	1.380 (2)	C18—H18A	0.9600
C8—H8	0.9300	C18—H18B	0.9600
C9—O2	1.3054 (19)	C18—H18C	0.9600
C9—C10	1.474 (2)	C19—H19A	0.9600
C10—C15	1.383 (2)	C19—H19B	0.9600
C10—C11	1.387 (2)	C19—H19C	0.9600
C11—C12	1.375 (2)	O2—H2A	1.16 (2)
			
F1—C1—C2	118.81 (15)	C14—C13—C12	115.83 (14)
F1—C1—C6	118.39 (16)	C14—C13—C16	122.26 (14)
C2—C1—C6	122.81 (16)	C12—C13—C16	121.89 (14)
C1—C2—C3	118.32 (15)	C15—C14—C13	122.32 (15)
C1—C2—H2	120.8	C15—C14—H14	118.8
C3—C2—H2	120.8	C13—C14—H14	118.8
C2—C3—C4	121.11 (16)	C14—C15—C10	120.86 (15)
C2—C3—H3	119.4	C14—C15—H15	119.6
C4—C3—H3	119.4	C10—C15—H15	119.6
C5—C4—C3	118.25 (14)	C19—C16—C13	111.57 (14)
C5—C4—C7	123.03 (13)	C19—C16—C18	109.51 (15)
C3—C4—C7	118.72 (14)	C13—C16—C18	107.85 (13)
C6—C5—C4	121.43 (14)	C19—C16—C17	108.31 (15)
C6—C5—H5	119.3	C13—C16—C17	111.03 (13)
C4—C5—H5	119.3	C18—C16—C17	108.51 (15)
C1—C6—C5	118.07 (16)	C16—C17—H17A	109.5
C1—C6—H6	121.0	C16—C17—H17B	109.5
C5—C6—H6	121.0	H17A—C17—H17B	109.5
O1—C7—C8	120.13 (14)	C16—C17—H17C	109.5
O1—C7—C4	117.12 (13)	H17A—C17—H17C	109.5
C8—C7—C4	122.71 (14)	H17B—C17—H17C	109.5
C9—C8—C7	121.11 (15)	C16—C18—H18A	109.5
C9—C8—H8	119.4	C16—C18—H18B	109.5
C7—C8—H8	119.4	H18A—C18—H18B	109.5
O2—C9—C8	120.78 (14)	C16—C18—H18C	109.5
O2—C9—C10	115.86 (14)	H18A—C18—H18C	109.5
C8—C9—C10	123.32 (15)	H18B—C18—H18C	109.5
C15—C10—C11	117.77 (15)	C16—C19—H19A	109.5
C15—C10—C9	122.06 (14)	C16—C19—H19B	109.5
C11—C10—C9	120.17 (15)	H19A—C19—H19B	109.5
C12—C11—C10	120.64 (15)	C16—C19—H19C	109.5
C12—C11—H11	119.7	H19A—C19—H19C	109.5
C10—C11—H11	119.7	H19B—C19—H19C	109.5
C11—C12—C13	122.56 (15)	C7—O1—H2A	101.2 (10)
C11—C12—H12	118.7	C7—O1—H2A	101.2 (10)
C13—C12—H12	118.7	C9—O2—H2A	102.1 (11)
			
F1—C1—C2—C3	178.96 (17)	O2—C9—C10—C11	29.4 (2)
C6—C1—C2—C3	−0.5 (3)	C8—C9—C10—C11	−148.40 (16)
C1—C2—C3—C4	−0.5 (3)	C15—C10—C11—C12	−1.3 (3)
C2—C3—C4—C5	1.3 (3)	C9—C10—C11—C12	178.87 (15)
C2—C3—C4—C7	−178.35 (16)	C10—C11—C12—C13	1.2 (3)
C3—C4—C5—C6	−1.2 (2)	C11—C12—C13—C14	0.0 (3)
C7—C4—C5—C6	178.49 (15)	C11—C12—C13—C16	178.28 (15)
F1—C1—C6—C5	−178.81 (15)	C12—C13—C14—C15	−0.9 (3)
C2—C1—C6—C5	0.6 (3)	C16—C13—C14—C15	−179.20 (15)
C4—C5—C6—C1	0.2 (3)	C13—C14—C15—C10	0.7 (3)
C5—C4—C7—O1	172.32 (15)	C11—C10—C15—C14	0.4 (3)
C3—C4—C7—O1	−8.0 (2)	C9—C10—C15—C14	−179.79 (15)
C5—C4—C7—C8	−10.2 (2)	C14—C13—C16—C19	−27.3 (2)
C3—C4—C7—C8	169.45 (15)	C12—C13—C16—C19	154.50 (16)
O1—C7—C8—C9	3.2 (2)	C14—C13—C16—C18	93.01 (19)
C4—C7—C8—C9	−174.20 (14)	C12—C13—C16—C18	−85.19 (19)
C7—C8—C9—O2	−1.8 (2)	C14—C13—C16—C17	−148.23 (16)
C7—C8—C9—C10	175.89 (14)	C12—C13—C16—C17	33.6 (2)
O2—C9—C10—C15	−150.37 (16)	C8—C7—O1—H2A	−3.3 (9)
C8—C9—C10—C15	31.8 (2)	C4—C7—O1—H2A	174.2 (9)

### Hydrogen-bond geometry (Å, °)

**Table:**

*D*—H···*A*	*D*—H	H···*A*	*D*···*A*	*D*—H···*A*
O2—H2A···O1	1.16 (2)	1.38 (2)	2.4720 (16)	154 (2)
